# Ki-67 expression predicts biochemical recurrence after radical prostatectomy in the setting of positive surgical margins

**DOI:** 10.1186/s12894-018-0330-y

**Published:** 2018-03-05

**Authors:** Mohammed Shahait, Samer Nassif, Hani Tamim, Deborah Mukherji, Maya Hijazi, Marwan El Sabban, Raja Khauli, Muhammad Bulbul, Wassim Abou Kheir, Albert El Hajj

**Affiliations:** 1 Division of Urology, Faculty of Medicine, American University of Beirut Medical Center, P.O. Box 11-0236, Riad El-Solh, Beirut, 1107 2020 Lebanon; 2 Department of Pathology, Faculty of Medicine, American University of Beirut Medical Center, P.O. Box 11-0236, Riad El-Solh, Beirut, 1107 2020 Lebanon; 3 Department of Internal Medicine, Faculty of Medicine, American University of Beirut Medical Center, P.O. Box 11-0236, Riad El-Solh, Beirut, 1107 2020 Lebanon; 4 Department of Physiology, Faculty of Medicine, American University of Beirut Medical Center, P.O. Box 11-0236, Riad El-Solh, Beirut, 1107 2020 Lebanon

**Keywords:** Ki-67, Positive surgical margin, Radical prostatectomy, Biochemical recurrence, Prostate cancer

## Abstract

**Background:**

Positive surgical margin (PSM) is a predictor of biochemical recurrence (BCR) following radical prostatectomy (RP). Attempts to stratify PSM based on linear length, Gleason score, location and number have failed to add to predictive models using margin status alone. We evaluated the prognostic significance of Ki-67 expression in this setting.

**Methods:**

Immunohistochemical staining for Ki-67 was done on prostatectomy specimens from 117 patients who had a PSM. Ki67 expression was measured at the margin and in the index lesion. Patients were dichotomized based on Ki-67 expression into three groups. Group 1 with no Ki-67 expression, Group 2 with Ki-67 ≤ 2%, and Group 3 with Ki-67 ≥ 3%.

To eliminate the impact of the adjuvant treatment (AT) on the outcome, data were analyzed by the Cox proportional hazards in which AT was Considered as a time-dependent covariate.

**Results:**

The discordance rate of Ki-67 expression between matched index lesion and margin specimens was 44/117 (37.6%). There was a trend for higher risk of BCR (HR:2.06, (0.97–4.43), *P* = 0.06) in patients expressing high Ki67 at the surgical margin although this was not statistically significant. However High Ki-67 expression in the index lesion was an independent predictive factor for BCR in this subset of patients. (HR:4, (1.64–9.80), *P* = 0.002).

**Conclusion:**

High Ki67 expression in the index prostate cancer lesion is an independent predictor of BCR in patients with positive surgical margin following radical prostatectomy. Our findings need to be validated in a larger cohort.

## Background

Prostate cancer is the second most common cancer to affect men worldwide, with an estimate 1.1 million new cases and 307,000 deaths in 2012 [1]. Radical prostatectomy (RP) is still the most common treatment for localized prostate cancer and has benefited from several refinements in surgical technique and technological advancements. A true measure of the oncological quality of RP remains the positive surgical margin which is considered an adverse prognostic feature that can predict biochemical recurrence (BCR). Many attempts at risk stratification of the positive margins have failed. Neither the number nor the sites of positive margins were found to have a significant impact on PSA recurrence [2]. More recently, Udo et al. showed that the linear length of positive surgical margins (PSM) in millimeters (LLOM) and highest Gleason grade or score at PSM are associated with progression. However, sub-categorization of surgical margins based on these parameters failed to add to predictive models using margin status alone [3].

Ki67-LI is a proliferation marker that is determined via a rapid, cheap and simple immunohistochemical method. The Ki67-LI, measured using MIB-1 antibody provides an estimate of the growth fraction of the tumor. Although not strongly expressed in prostate cancer cells, several studies have shown its potential role in predicting BCR and even prostate cancer-specific mortality. High Ki67-LI was an independent predictor of increased disease specific mortality and biochemical recurrence in primarily intermediate-risk prostate cancer patients treated with RT or Radical prostatectomy [4, 5]. More recently, Tollefson et al. found that each 1% increase in Ki-67 expression was associated with a 12% increased risk of prostate cancer-specific death [6].

The aim of our study was to measure the Ki67 at the surgical margin and index lesion in a radical prostatectomy series and correlate Ki-67 expression with BCR.

## Methods

### Patient population:

All studies were undertaken with the approval and oversight of the Institutional Review Board for the Protection of Human Subjects at the American University of Beirut. The consent for tissue processing was waived by the IRB. The study comprised of 117 patients who underwent open radical prostatectomy between 1998 and 2012 and had a positive surgical margin at the final pathology.

No patient had clinical lymph node involvement or distant metastatic disease at the time of prostatectomy. Exclusion criteria were patients with pathologic lymph node involvement, patients treated with neoadjuvant therapy, patients with persistent PSA level, and those for whom original pathology slides were unavailable.

### Tissue processing

For every patient, all slides from the prostatectomy specimen were reviewed separately by two pathologists to identify the sections/block showing a positive surgical margin, and to re-assess the Gleason score based on the most up-to-date Gleason scoring method. In cases where margins were positive multifocally, sections harboring the largest focus of tumor were selected. The lengths of the positive margin, as well as the Ki-67 proliferation index, were noted for all cases. The Ki-67 index was also assessed independently by the same two pathologists using the following method: 1-the tumor area was screened to identify the foci with the highest nuclear staining; 2-these foci were divided into quadrants and examined at high power magnification (400×); 3- tumor nuclei were counted in each quadrant, and the percentage of positive tumor nuclei was determined. For each case with a positive margin, the Ki-67 index was assessed at the margin and in the index tumor.

### Immunohistochemistry

Immunostaining for Ki-67 was performed using the DAKO MIB-1 antibody. Staining was performed on the Ventana immunostainer using protocols established by the manufacturer and approved for clinical practice. With each staining run, positive control tissues were used to ensure adequate staining performance.

### Follow-up

The 117 patients were followed from 10 to 106 months with a median of 48 months (mean 58.2 months). Biochemical recurrence was defined as a PSA greater than 0.2 ng/ml confirmed on two consecutive PSA examinations. A total of 62 patients received adjuvant radiotherapy (AT) with concomitant hormonal therapy (6–18 months) before evidence of biochemical or clinical recurrence.

### Statistical analysis

Categorical variables were compared by the chi-square test. Cox proportional hazards regression analysis was used to test the association of various pathological and clinical features with recurrence. To eliminate the impact of the adjuvant treatment (AT) on the outcome, data were analyzed by the Cox proportional hazards in which AT was considered as a time-dependent covariate as described previously by Swindle et al. [7].

**Fig. 1:**
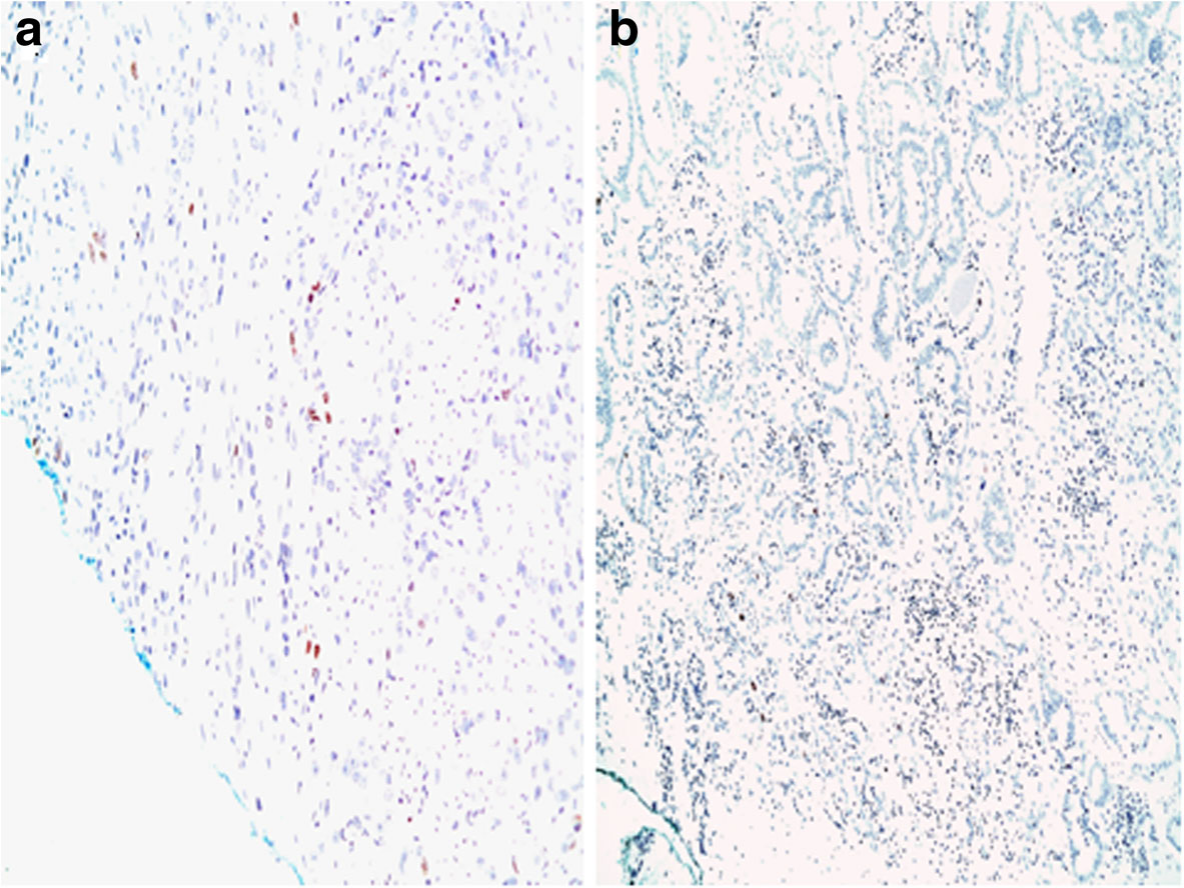
Ki-67 expression at the margin. **a**: Group 2 with Ki-67 expression less than or equal to 2%. **b**: Group 3 with Ki-67 expression ≥3%

Patients were stratified based on Ki-67 expression into three groups based on the distribution of the original variables. Group 1 with no Ki-67 expression, Group 2 with Ki-67 expression less than or equal to 2%, and Group 3 with Ki-67 expression ≥3% (Fig. 1).

Univariable and multivariable Cox regression models were carried out to determine independent predictors of PSA failure in patients with positive surgical margin. The variable that was significant at *p* < 0.2 at the univariable level was entered into the multivariable model. Age of patients, PSA, Gleason score, SVI, and EPE, were forced into the multivariable model. Hazard ratios (HR) and their 95% confidence intervals (CI) were reported.

## Results

### Clinicopathological parameters

Mean age at radical prostatectomy was 62.01 years (± 6.06). Sixteen patients (13.68%) had a Gleason score < =6, 79 (67.52%) had a Gleason score equal to 7 and 22 patients (18.8%) had a Gleason score > =8. Fifty-two patients (44.4%) had an extraprostatic extension, 23 patients (19.6%) had a seminal vesicle involvement. Adjuvant treatment was administered in 62 (52.99%) of the cases. Fifty-seven patients of the 117 (48.7%) developed biochemical recurrence during the follow-up period (Table 1).

**Table 1:** Summary of the clinical and pathological data

			PSA failure		
		Total *N* = 117	Negative *N* = 60	Positive *N* = 57	*p*-value
Age	Mean (±SD)	62.01 ± 6.06	62.35 ± 5.98	61.65 ± 5.21	0.53
PSA-PRE surgery	0–10	69 (61.1)	42 (72.4)	27 (49.1)	0.04
	10–20	31 (27.4)	11 (19.0)	20 (36.4)	
	> 20	13 (11.5)	5 (8.6)	8 (14.6)	
GS	6	16 (13.68)	9 (15.00)	7 (12.28)	0.39
	7	79 (67.52)	43 (71.67)	36 (63.16)	
	8	17 (14.53)	7 (11.67)	10 (17.54)	
	9	5 (4.27)	1 (1.67)	4 (7.02)	
Tumor volume	Mean (±SD)	26.52 ± 20.80	26.29 ± 19.30	26.78 ± 22.62	0.90
EPE	No	65 (55.56)	37 (61.67)	28 (49.12)	0.17
	Yes	52 (44.44)	23 (38.33)	29 (50.88)	
SVI	No	94 (80.34)	49 (81.67)	45 (78.95)	0.71
	Yes	23 (19.66)	11 (18.33)	12 (21.05)	
Linear Length of the Margin	< 3 mm	48 (41.38)	28 (46.67)	20 (35.71)	0.23
	> = 3 mm	68 (58.62)	32 (53.33)	36 (64.29)	
KI67 at the Margin	1 (no expression)	64 (54.70)	31 (51.67)	33 (57.89)	0.42
	2 (weak expression)	31 (26.50)	19 (31.67)	12 (21.05)	
	3 (strong expression)	22 (18.80)	10 (16.67)	12 (21.05)	
Number of Margin	Single	55 (47.01)	30 (50.00)	25 (43.86)	0.51
	Multiple	62 (52.99)	30 (50.00)	32 (56.14)	
KI67-deep tumor	1 (no expression)	92 (78.63)	49 (81.67)	43 (75.44)	0.71
	2 (weak expression)	11 (9.40)	5 (8.33)	6 (10.53)	
	3 (strong expression)	14 (11.97)	6 (10.00)	8 (14.04)	
Adjuvant treatment	No	55 (47.01)	31 (51.67)	24 (42.11)	0.30
	Yes	62 (52.99)	29 (48.33)	33 (57.89)	

### Margin status

Of the patients 62 (52.99%) had multiple margins and the linear length of the margin (LLOM) was greater than 3 mm in 68 (58.62%). Ki-67 was expressed in 53 of 117 (45.3%) of the surgical margin.

### Immunohistochemistry

**Fig. 2:**
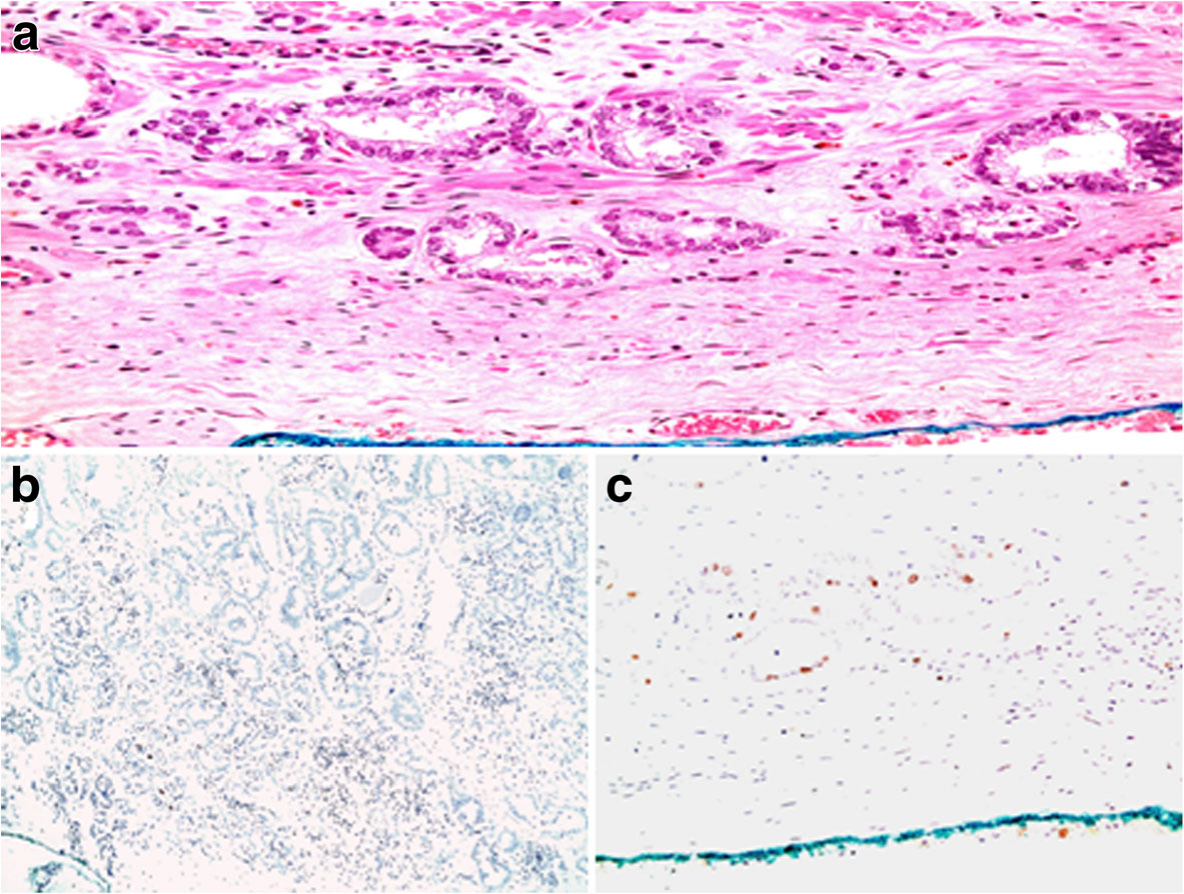
Ki-67 expression at the margin and at the index lesion. **a**: Prostate Adenocarcinoma at the inked margin. **b**: High Ki-67 expression at the margin. **c**: Low Ki-67 expression at the index lesion

Ki-67 was expressed in 25 of 117 (21%) of the index lesion and 53 of 117 (45%) of the matched margins. The discordance rate of Ki-67 expression between matched index lesion and margin specimens was 44/117 (37.6%) with a trend of higher Ki-67 expression at the margin compared to the index lesion (Fig. 2). The mean Ki-67 expression at the margin was 1.32% (±2.16).

### Cox proportional hazards regression analysis

Higher Ki-67 expression at the margin showed a trend toward significant association with higher risk of BCR (HR:2.06, (0.97–4.43), *P* = 0.06). On the other hand, LLOM and number of the margin were not correlated with biochemical recurrence (Table 2).

**Table 2:** Predictors of biochemical recurrence on multivariable cox regression analysis

	HR	95% CI	*P* value
Ki 67 in deep tumor			
Group 1	Reference		
Group 2	1.41	(0.57–3.52)	0.46
Group 3	4	(1.64–9.80)	0.002
Ki 67 at margin			
Group 1	Reference		
Group 2	1.12	(0.55–2.31)	0.75
Group 3	2.08	(0.97–4.43)	0.06
Length of the margin			
< 3 mm	Reference		
> =3 mm	1.28	(0.70–2.37)	0.42
Number of margin			
Single margin	Reference		
Multiple margins	0.83	(0.45–1.51)	0.54

High Ki-67 expression in the deep tumor was an independent predictor of biochemical recurrence (HR:4, (1.64–9.80), *P* = 0.002).

### Discussion

In this cohort of patients with positive surgical margin after radical prostatectomy, our results show that high Ki- 67 expression in the deep tumor was a significant predictor of BCR and high Ki-67 expression at the margin showed a trend toward significant association with BCR. We also found that there is no correlation between LLOM and extent of margin on the biochemical recurrence.

PSM is an established adverse pathological feature correlated with biochemical progression [7]. Indeed, this correlation has impacted urologist practice by utilizing adjuvant treatment in patients with PSM; Grossfeld et al. using the CaPSURE database found that patients with a positive margin were much more likely to receive adjuvant treatment (p 0.0011) [2].BCR rate in our cohort is in line with previously observed rate in PSM patients (32%–74%) [8, 9]. In the observation arm of the European Organisation for Research and Treatment of Cancer 22,911, which compared the administration of AT versus patient observation, 41% of the patients were disease-free at five years. Therefore, it is evident that there is overtreatment of patients with positive surgical margin, which might have an adverse impact on patient care, given that the use of AT is not without acute and late gastrointestinal and genitourinary toxicities [10].

Several studies attempted to classify surgical margins based on different features, such as length of the margin, the extent of the margin, and Gleason score at the margin. Sofer et al. found that there is no correlation between surgical margin site and biochemical progression. Moreover, he demonstrated that there was no statistically significant difference in time to progression between patients with single margin and patients with multiple margins [11]. Additionally, Epstein et al. failed to demonstrate any difference in the progression between patients with single margin and those with multiple margins [12]. In the current study, there was no statistically significant difference in prognosis between men with a single site of a positive margin or multiple sites, supporting results from previous studies. However, while LLOM more than 3 mm have been shown to confer a worse prognosis compared to margins < 3 mm, we were unable to validate this finding in our cohort [13, 14].

Ki-67 is an established marker of cell proliferation, which has been previously studied in prostate cancer and was correlated with biochemical progression, prostate cancer-specific survival and overall survival [4, 15, 16]. No study to our knowledge has examined the importance of Ki-67 expression in the setting of positive surgical margins.

Mesko et al. demonstrated a significant heterogeneity in intraprostatic and intralesional expression of Ki-67 [17]. We found that there is high heterogeneity of Ki-67 expression between the index lesion and the margin, with a discordance rate of 37.6%. This has stirred us to measure ki-67 expression at the margin and the index lesion.

Intriguingly, the mean Ki-67 expression in our cohort was 0.5% (±1.32) which is lower than the levels reported in the literature [17, 18]. There was no significant difference in overall Ki-67 proliferation values when comparing stains performed on old tissue blocks (more than five years old) to stains carried out on more recent blocks. The difference between our results and previously published results is unclear at this point, but could be attributed to the racial differences in the prostate cancer biology [19–22].

In the current study we assessed the significance of Ki-67 at margin accounting for the patients who received AT. Our study has shown that there was a trend towards biochemical recurrence in PSM patients with higher Ki-67 expression at the margin however statistical significance was not attained (HR 2.06, CI 0.97–4.43 *P* = 0.06). On the other hand, Ki67 expression in the index tumor was an independent predictor of BCR (*p* = 0.002). This finding may help in the discussion of post-prostatectomy treatment for patients with PSM.

We acknowledge that the present study has some limitations that warrant discussion. First, the data analyzed are retrospective and included patients operated on before the 2005 International Society of Urological Pathology modified Gleason score system. For this reason all pathology slides were reviewed by two expert pathologists and the Gleason score was revised according to the new classification. Second, there are inherent limitations to the reliability and reproducibility of the immunohistochemical techniques.

## Conclusion

Ki67 expression at the margin is a potential tool to predict biochemical recurrence in patients with positive surgical margins following radical prostatectomy. There is a need to validate this finding in a larger prospective trial and determine reference Ki-67 values. In the era of genetic testing, this cost effective and simple marker can be an additional tool to determine the optimal care in this subset of patients.

## Abbreviations

AT: Adjuvant Therapy
BCR: Biochemical recurrence
CI: Confidence intervals
EPE: Extra-prostatic extension
GS: Gleason score
HR: Hazard ratios
LLOM: Linear length of the margin
PSA: Prostate specific antigen
PSM: Positive surgical margin
RP: Radical prostatectomy
SVI: Seminal Vesicle Invasion
